# Bariatric Surgery and Precision Nutrition

**DOI:** 10.3390/nu9090974

**Published:** 2017-09-06

**Authors:** Carolina F. Nicoletti, Cristiana Cortes-Oliveira, Marcela A. S. Pinhel, Carla B. Nonino

**Affiliations:** 1 Internal Medicine Department, Ribeirão Preto Medical School, University of São Paulo, Ribeirão Preto, São Paulo 14049-900, Brazil; carol_nicolettif@yahoo.com.br (C.F.N.); cristiana.cortes@outlook.com (C.C.-O.); marcelapinhel@yahoo.com.br (M.A.S.P.); 2 Molecular Biology Department, São Jose do Rio Preto Medical School, São José do Rio Preto, São Paulo 15090-000, Brazil

**Keywords:** obesity, bariatric surgery, gene, polymorphism, gene expression, epigenetics, DNA methylation, microbiota, biomarkers

## Abstract

This review provides a literature overview of new findings relating nutritional genomics and bariatric surgery. It also describes the importance of nutritional genomics concepts in personalized bariatric management. It includes a discussion of the potential role bariatric surgery plays in altering the three pillars of nutritional genomics: nutrigenetics, nutrigenomics, and epigenetics. We present studies that show the effect of each patient’s genetic and epigenetic variables on the response to surgical weight loss treatment. We include investigations that demonstrate the association of single nucleotide polymorphisms with obesity phenotypes and their influence on weight loss after bariatric surgery. We also present reports on how significant weight loss induced by bariatric surgery impacts telomere length, and we discuss studies on the existence of an epigenetic signature associated with surgery outcomes and specific gene methylation profile, which may help to predict weight loss after a surgical procedure. Finally, we show articles which evidence that bariatric surgery may affect expression of numerous genes involved in different metabolic pathways and consequently induce functional and taxonomic changes in gut microbial communities. The role nutritional genomics plays in responses to weight loss after bariatric surgery is evident. Better understanding of the molecular pathways involved in this process is necessary for successful weight management and maintenance.

## 1. Introduction

Bariatric surgery, including gastric bypass, has emerged as the most effective strategy to treat obesity and its associated comorbidities [1,2]. Non-surgical treatments generally fail to provide substantial and long-term weight loss in severe obesity cases [3]. Every year, about 500,000 bariatric surgical procedures are performed worldwide; sleeve gastrectomy (SG, 49%) and Roux-en Y gastric bypass procedure (RYGB, 43%) are the most commonly performed techniques [4,5,6].

Long-term excess body weight reduction is a major goal of bariatric surgery. Excess weight loss is about 62%, 68%, and 48% for RYGB, vertical-banded gastroplasty (VBG), and laparoscopic adjustable gastric banding (LAGB), respectively [7]. Despite the positive effects of bariatric surgery, weight regain; that is, recovery of 10 to 20% of the minimum weight achieved by the patient [8], occurs in between 30% and 50% of the patients at the late postoperative period (between one and a half and two years after the surgical procedure) [9,10].

Just as genetic and epigenetic signatures influence the obesity phenotype [11], genetics recognizably underlies weight loss percentage, resistance, and maintenance after surgical treatment [11,12]. Different surgical techniques (restrictive, malabsortive, or a combination of both) [13] and genetic background [14] account for the wide variation in responses to bariatric surgery.

In the last decades, many efforts have been made to understand the variations in inter-individual responses to the same obesity treatment strategy. In this context, “omics” sciences such as genomics, transcriptomics, proteomics, metabolomics, microbiomics, and epigenomics have emerged. Together, these sciences establish genomic nutrition [15]. Genetic variation among individuals underlies the variety of physiological responses in the same environment and explains why some individuals are more likely to gain/lose weight than others in the same environmental conditions [16], including weight gain/loss after bariatric surgery. However, the complex interactions between nutrients and genes have not been fully elucidated [17].

**Figure 1:**
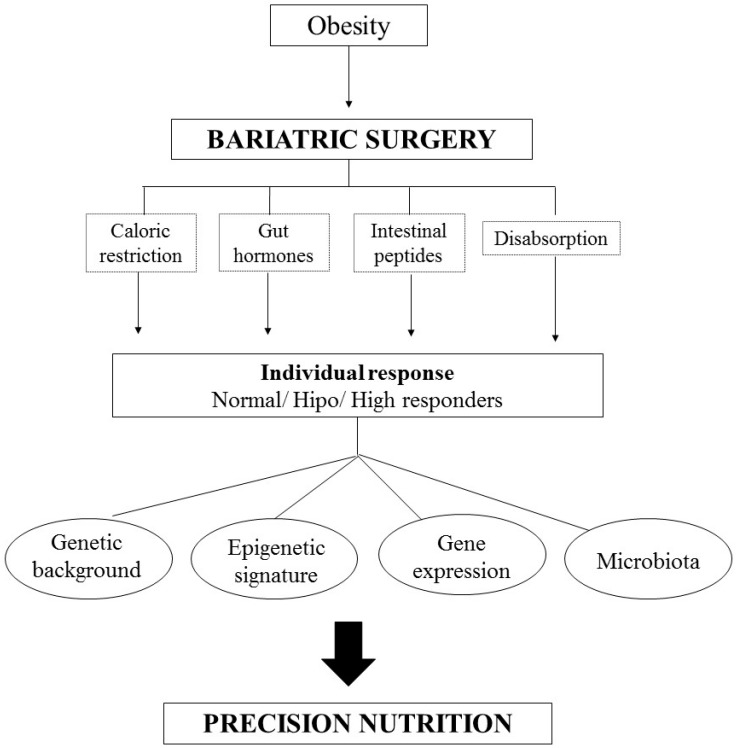
Algorithm of Personalized Nutrition in Bariatric Surgery. The individual responses of the surgery are due, in addition to the caloric restriction, alterations of the gut hormones and the malabsorption process, of the individuals’ genetic information, as well as of the epigenetic signature, modifications in the gene expression and the microbiota.

In this scenario, precision nutrition in bariatric surgery is an important tool in personalized medicine and may target specific guidelines based on interindividual differences (Figure 1). In this paper, we summarize the main literature findings relating nutritional genomics and bariatric surgery.

## 2. Genetic Background and Bariatric Surgery Management

Nutrigenetics studies inborn genetic variants that predict an individual’s risk for disease and explains the individual’s nutritional requirements and nutrient absorption, metabolism, and excretion [18,19]. In this sense, it is possible to affirm that genetic factors partially determine susceptibility to obesity, and that an obesogenic environment is necessary for phenotypic obesity expression. Therefore, despite new evidence that genetics influences obesity, it is necessary to consider that biological and psychosocial factors interact in a complex way [19].

In the context of obesity treatment, different types of patients exist. Obese patients may be classified as normo-responders, hypo-responders, or hyper-responders, depending on their phenotypic response to diet or surgical treatment. This indicates that not only the environment but also genetic variations account for successful weight-loss therapy [20,21]. Several genes as well as single nucleotide polymorphisms (SNPs) are associated with obesity phenotypes and weight loss after bariatric surgery [21,22,23,24,25].

Novais et al. (2016) [24] evidenced that the *5-HT2C* gene polymorphism (rs3813929) is associated with a greater percentage of excess body weight after RYGB. Two SNPs in the *UCP2* gene (Ala55Pro and −866G > A) are considered as biomarkers of weight loss after bariatric surgery [22]. When Seip et al. (2016) [26] evaluated 330 SNPs of genes involved in metabolic regulation they identified many genes that could be potential markers to discriminate changes in body mass index (BMI) one year after surgical intervention (LAGB or RYGB). Other authors suggested that polymorphisms, like the SNP of the preproghrelin gene (rs696217), could mark a successful weight loss outcome [27]. Table 1 lists recent studies that associate SNPs and bariatric surgery outcomes.

Algorithms that predict the chances of treatment failure or that even help to identify guidelines to prevent body weight recovery are critical to obesity management [24]. Nicoletti et al. (2016) [30] proposed a genetic predisposition score to estimate the contribution of seven obesity-related polymorphisms to the weight loss process after one year of RYGB. The authors found that lower score is associated with higher weight and BMI values, which shows that the higher the number of effect alleles, the lower the severity of obesity and the better the metabolic outcomes after the surgical procedure. Moreover, weight regain in the late postoperative period is more frequent and occurs sooner in individuals that carries a polymorphism in the *FTO* gene (rs9939609) [32].

It is noteworthy that the genetic variants reported in the literature are located in genes associated with obesity development per se, thermogenesis, adipogenisis, and eating behavior/appetite control. However, the mechanisms through which these variants influence weight loss are largely unknown. Some authors investigated variants of genes associated with eating behavior in obese individuals before and after bariatric surgery. Bandstein et al. (2016) [31] demonstrated that the rs4846567 SNP in lysophospholipase-like 1 gene is associated with the hunger score, a factor that can determine therapeutic success. Likewise, a haplotype of the *UCP2* gene is associated with dietary consumption after RYGB. Nicoletti et al. (2016) [30] showed that carriers of at least one rare haplotype present greater energy and carbohydrate intake [30].

Located at the end of eukaryotic chromosomes, telomeres are special structures characterized by a TTAGGG sequence [33], which ensures stable genetic material inheritance [34]. Telomeres shorten progressively at each cellular division, and they can serve as a cellular aging marker [35]. Several large studies show that telomere length (TL), adiposity, and BMI are inversely associated [36,37].

Knowing that oxidative stress and chronic inflammation are related to weight gain, and that obesity is associated with telomere shortening [38,39], studies associate shorter telomeres with obesity comorbidities such as diabetes and hypertension [40,41]. On the other hand, published works show that diet components (e.g., fiber) and caloric restriction with significant weight loss influence TL and possibly have a preventive effect on telomere shortening [42,43].

The fact that weight loss induced by bariatric surgery does not restore short TL after one year was one of the first signs that bariatric surgery impact TL [43]. Formich et al. (2014) [44] discussed that the immediate postoperative period is characterized by a catabolic state, which accelerates telomere erosion [44,45]. In contrast, Dersham et al. (2017) [37] evaluated subjects between three and five years after gastric bypass. The latter authors observed increased TL and emphasized that significant lengthening occurs in patients with the shortest baseline TL. Nevertheless, TL does not correlate with weight loss percentage. In the late postoperative period (10 years), some authors also verified increased TL [46]. These changes in TL may stem from weight loss itself and from an improved metabolic condition, which reduce telomere attrition [46]. Interestingly, a study that investigated subjects before and six months after bioenteric intragastric balloon found that individuals who present greater weight loss show greater telomere lengthening [47].

Further studies are necessary to assess and to establish interactions between these genetic variants and bariatric surgery outcome, so that biomarkers can be determined for more personalized weight loss management.

## 3. Epigenetic Signatures Related to Bariatric Surgery Outcomes

DNA is a highly dynamic biomolecule, which is reflected in its diverse and complex regulation [48]. Epigenetics is defined as an inheritable process during which reversible changes in the chromatin structure take place without involving the underlying DNA sequence, to impact transcriptional control and cellular function [49,50]. Epigenetic alterations include DNA methylation, histone modification (such as histone acetylation and methylation), and noncoding RNAs (e.g., microRNAs) [51,52,53].

In this context, epigenome dysregulation may modify an individual’s phenotype and lead to numerous chronic diseases, like obesity [54,55]. In a recent guide paper, authors discussed interactions among dietary components and epigenetic alterations involved in disease risk [56]. For example, high-fat and high-sugar diet is related to leptin and fatty acid synthase methylation, which consequently contributes to the obesity phenotype in Wistar rats [57,58]. Other studies describe the role epigenetic markers play in the anthropometric and metabolic outcomes of obesity treatment [59,60]. Methylation patterns of appetite-regulatory genes are related to weight loss and regain after eight-week nutritional intervention [61]. Indeed, Nicoletti et al. (2016) [62] showed that DNA methylation patterns behave differently according to the adopted weight loss strategies.

In the bariatric surgery context, genome-wide DNA methylation analysis shows that weight loss is associated with changes in methylation at CpG and exonic regions close to transcription start sites [63]. Numerous mechanisms are likely to contribute to metabolic improvements after RYGB surgery; for example, restricted calorie ingestion, rapid influx of undigested complex nutrients, and altered gut and intestinal hormone secretion [64,65]. However, authors have recently reported that changes in DNA methylation could be another mechanism that contributes to the metabolic outcomes observed after gastric bypass and to postoperative metabolic homeostasis [63,66]. Table 2 summarizes the main recent studies that evaluate epigenetic modifications after bariatric surgery. Epigenetic changes related to bariatric surgery may be due to weight loss per se or to other factors related to the surgical procedure, such as the daily use of vitamin-mineral supplements and alterations in hormone secretion and dietary intake after the procedure [62].

Furthermore, a specific gene methylation profile can be used to predict weight loss after RYGB. Nicoletti et al. (2016) [62] evidenced that high responders have lower *SERPINE-1* methylation levels six months after RYGB. There is evidence that the methylation profiles in promoter gene regions of postoperative obese patients presenting weight loss become similar to the methylation profiles of normal-weight individuals [72]. Barres et al. (2013) [63] compared obese women before and six months after RYGB. These authors found increased and decreased promoter methylation of pyruvate dehydrogenase kinase, isoenzyme 4 *(PDK4)* and proliferator-activated receptor g coactivator-1 a (*PGC-1a*), respectively, and observed that surgery normalized this pattern to the levels in control women [63]. Methylation profile normalization occurs at the same time that metabolic parameters such as fasting glucose, total cholesterol, and triglycerides concentrations normalize [63].

Remarkably, studies demonstrate that bariatric surgery promotes durable and detectable changes in subsequent offspring methylome and transcriptome [73]. When Guénarda et al. (2013) [73] compared methylation profiles in siblings born before and after the surgical procedure, these authors found that 3% of the probes are differentially methylated, and they identified differences in genes that underlie improved cardiometabolic risk profile. Moreover, the sperm methylome is changed in morbidly obese men submitted to RYGB, and these gametic epigenetic modifications modify the metabolic profile [74].

DNA methylome studies hold enormous promise for personalized medicine and nutrition, but technological challenges like cost-effective sample analysis impair such studies [75].

## 4. Bariatric Surgery and Gene Expression Profile

Nutrigenomics is the science that studies how nutrients and food components influence gene expression profile [76]. This science also examines how a nutritional strategy affects gene expression and uses the gene expression pattern as a tool to predict responsiveness to nutritional treatments [56,77]. Dietary caloric restriction is sufficient to alter the expression of different genes related to resting energy expenditure and lipid metabolism and contributes to the development of strategies for obesity and weight control [78,79,80].

In this sense, bariatric surgery may affect the expression of various genes involved in different metabolic pathways [80]. Table 3 depicts some interactions between bariatric surgery and the gene expression profile. A recent study on whole transcriptome analysis evidenced that about 1366 genes are differentially expressed (1188 upregulated and 178 downregulated genes) in the postoperative period as compared to the preoperative period of RYGB, and that these genes are associated with gene transcription, lipid and energetic metabolism, immunological processes, cell differentiation, oxidative stress, substrate oxidation, and adipocyte differentiation [81].

Ortega et al. (2016) [83] observed that acutely postoperative RYGB changes insulin receptor substrate 1 (*IRS1*) expression in the subcutaneous adipose tissue. Concomitantly, RYGB increases expression of inflammatory (interleukin (IL) 6 (*IL-6*), *IL-8*, and tumor necrosis factor alpha (*TNF-alpha*)) and lipogenic (lipopolysaccharide binding protein, *LBP*) genes. Moreover, a recent study showed that gene expression patterns in subcutaneous adipose tissue one year postoperatively are characteristic of a reduced inflammatory profile [86]. In contrast, cytokine expression in adipose tissue does not change one and 12 months after the surgical procedure [89].

Expression of the gene*UCP2*, a gene that participates in thermogenesis and body weight regulation, affects weight loss after bariatric surgery. A study conducted with obese women revealed increased *UCP2* expression six months after the surgical procedure and a positive association between baseline gene expression and weight loss percentage [85].

Eutrophic individuals and obese patients have different gene expression profile, and bariatric surgery modulates this default [87]. Knowledge of the metabolic pathways affected by this surgical procedure is important when bariatric surgery is indicated and ensures successful obesity treatment [85].

## 5. The Role of Bariatric Surgery on Microbiota

According to several studies, bariatric surgery as a strategy to achieve weight loss plays a crucial part in functional and taxonomic changes observed in the gut microbial communities after surgery [90,91,92]. Gut microbiota is associated with an individual’s metabolic health, so it is essential that these microorganisms be taken into consideration during development of new personalized treatments and identification of biomarkers of different metabolic diseases [90,93]. Given that bariatric surgery elicits significant anatomical and physiological alterations in the gastrointestinal tract regardless of the surgical technique, there is growing interest in understanding intestinal microbiota modification and in establishing how these changes contribute to improving the metabolic profile and the weight loss process.

Liou et al. (2013) [92] evaluated germ-free mice that received intestinal microbiota from other animals that had undergone RYGB surgery. These authors found that the former animals had reduced diet caloric intake, increased resting energy expenditure, and higher fatty acid concentrations, which proved transmission of these characteristics. Moreover, Tremaroli et al. (2015) [91] verified that germ-free mice that received fecal microbiota collected from humans nine years after RYGB or SG had lower body fat accumulation (46% and 26%, respectively) two weeks after transplantation as compared to rats that received microbiota from obese individuals that had not undergone any surgical procedure. In addition, the test animals used more fat as energy substrate, but energy expenditure at rest remained unaltered.

In a more recent study, Palleja et al. (2017) [94] compared patients before and after bariatric surgery. These authors verified not only weight loss and improved glycemic profile, but also alterations in the intestinal microbiota, including changes in microbiota diversity and composition within three months after the surgical procedure. In addition, more than half of the altered microbiota species were maintained in the long term, which indicated that bariatric surgery could lead to rapid and sustained changes in the patients' gut microbiota.

Human feces microbial composition analysis showed that six main phyla are present therein: Bacteroidetes, Firmicutes, Proteobacteria, Actinobacteria, Fusobacteria, and Verrucomicrobia [95]. Some studies compared patients’ pre- and postoperative (RYGB) microbiota to non-operated control individuals’ microbiota, to detect alterations in intestinal bacteria after surgery; more specifically, increased Proteobacteria and Bacteroidetes and decreased Firmicutes (Table 4).

New studies aiming at better understanding the interactions between microbiota and obesity and the possible ways to modulate gut microbiota could benefit bariatric surgery patients in the future.

## 6. Conclusions

Surgical management of obesity requires understanding the genetic and epigenetic factors that play a crucial key role in obesity development and weight loss response. Given the concepts of nutritional genomics, defining a “nutrigenomic risk score” or a “nutrigenomic profile” for each individual may represent a novel therapeutic approach for the management of obese patients submitted to bariatric surgery. We believe that nutritional genomics will soon enable the delivery of precise nutrition recommendations to patients undergoing bariatric surgery, to provide high-risk individuals with personalized treatment and to prevent complications.

## Figures and Tables

**Table 1:** List of recent articles associating the outcomes of different types of bariatric surgery with single nucleotide polymorphisms (SNPs).

Genes	Polymorphism	Main Results	Surgery/Time	*N*° Patients	Ref.
*MC4R*	rs17782313	Women carrying this polymorphism present higher pre-surgical BMI and tend to maintain BMI > 35 kg/m^2^, which characterizes treatment failure	60 months of RYGB	217	[21]
*UCP2*	Ala55Val −866G > A	Mutated alleles (T and A) could be biomarkers of weight loss	12 months of RYGB	150	[22]
*FTO*	rs9930506	Weight percentage is significantly higher in carriers of the AG and GG genotypes	Six months of sleeve gastrectomy	11	[28]
*PNPLA3* *TM6SF2* *MBOAT7*	p.I148M p.E167E rs641738	Mutated allele might be associated with greater improvement of hepatic steatosis after bariatric surgery	One year of gastric bypass gastric sleeve	84	[29]
*5-HT2C*	rs3813929	The TT genotype predicts greater percentage of excess weight loss among female patients	12 months of RYGB	351	[24]
*FKBP5*	rs1360780	The T allele is associated with weight loss.	Bariatric surgery	42	[25]
*UCP2*	Ala55Val −866G > A	Patients with at least one rare allele for polymorphisms and with at least one rare allele for both polymorphisms together (haplotype) present greater energy and carbohydrate intake even after adjustment for gender, age, and weight.	12 months of RYGB	150	[30]
32 SNPs	-	The LYPLAL1 genotype is associated with different eating behavior and loss of extensive body weight	Two years of RYGB	251	[31]
330 SNPs	-	Information derived from patient DNA may be useful to predict surgical weight loss outcomes and to guide selection of surgical approach.	One year of RYGB or LAGB	161	[26]
*FTO*	rs9939609	Weight loss progresses differently in obese carriers of the FTO gene variant rs9939609 after bariatric surgery	Two months of RYGB	146	[32]

*MC4R*: melanocortin 4 receptor; *UCP2*: uncoupling protein 2; *FTO:* alpha-ketoglutarate-dependent dioxygenase or fat mass and obesity-associated protein; *PNPLA3*: patatin-like phospholipase domain containing 3; *TM6SF2*: transmembrane 6 superfamily member 2; *MBOAT7*: membrane-bound O-acyltransferase domain containing 7; *5-HT2C*: 5-hydroxytryptamine receptor 2C; *FKBP5*: FK506 binding protein 5; *n*: number of individuals; RYGB: Roux-en-Y gastric by-pass; LAGB: laparoscopic adjustable gastric banding; BMI: body mass index.

**Table 2:** Recent studies evaluating epigenetics modifications after bariatric surgery.

Target Gene	Type of Material	Modification Type	Surgery/Time	Ref.
*IL-6*	Whole blood	Decrease	six months after RYGB	[62]
*PDK4*, *IL-6*, and *TNF*	Whole blood	Increase	12 months after RYGB	[66]
*SCD-1*	Whole blood	Increase	six months after RYGB	[67]
*PGC-1a* and *PDK4*	Skeletal muscle	-	six months after RYGB	[63]
*ADK*	-	Decrease	six months after RYGB	[68]
*PTPRE*	Liver	Increase	-	[69]
*ETP*, *FOXP2*, *HDAC4* and *DNMT3B*	Adipose tissue	Decrease	-	[70]
Global *LINE-1*	Whole blood	Not modified	six months after RYGB	[71]

*IL-6*: interleukin 6; *PDK4:* pyruvate dehydrogenase kinase 4; *TNF*: tumor necrosis fator; *SCD-1*: stearoyl CoA desaturase-1; *PGC-1a*: proliferator-activated receptor g coactivator-1 a; *ADK*: adenosine kinase; *PTPRE*: protein tyrosine phosphatase, receptor type E; *ETP*: early T-cell precursor; *FOXP2*: forkhead box protein P2; *HDAC4*: histone deacetylase 4; *DNMT3B*: DNA methyltransferase 3 beta; *LINE-1*: long interspersed nuclear elements; RYGB: Roux-em Y gastric bypass.

**Table 3:** Some interactions between bariatric surgery and the gene expression profile.

Gene	Modification Type	Related Metabolic Pathways	Surgery/Time	Ref.
*GGT1*, *CAMP*, *DEFA1*, *LCN2*, *TP53*, *PDSS1*, *OLR1*, *CNTNAP5*, *DHCR24*, and *HHAT*	-	Lipid metabolism and obesity development	Six to twelve months after bariatric surgery (SG and RYGB)	[82]
*IL-6*, *IL-8*, and *TNF*-alpha *GLUT4*, *IRS1*, and adiponectin	Increase Decrease	Inflammation Glucose metabolism	Acutely postoperative RYGB	[83]
*CIDEA* Glutathione	Increase	Lipid droplet formation in the adipose tissue Glutathione metabolism	12 months after bariatric surgery (SG and RYGB)	[84]
*UCP2* *PLIN1*	Increase Unchanged	Thermogenesis Lipolysis	Six months after RYGB	[85]
*TNF*, *CASP3*	Increase	Inflammation	12 months after bariatric surgery	[86]
*Leptin* *PPARg1* *PPARg2*	Decrease Increase Unchanged	Insulin metabolism	12 months after RYGB	[87]
*UCP1* *UCP3*	Unchanged	Thermogenesis	Six months after RYGB	[88]

*GGT1*: gamma-glutamyltransferase; *CAMP*: cathelicidin antimicrobial peptide; *DEFA1*: defensin alpha 1; *LCN2*: lipocalin 2; *TP53:* tumor protein p53; *PDSS1*: decaprenyl diphosphate synthase subunit 1; *ORL1*: oxidized low-density lipoprotein receptor 1; *CNTNAP5*: contactin- associated protein like 5; *DHCR24*: 24-dehydrocholesterol reductase; *HHAT*: hedgehog acyltransferase; *IL-6*: interleukin 6; *IL-8*: interleukin 8; *TNF*: tumor necrosis factor; *GLUT4*: glucose transport; *IRS1*: insulin receptor substrate 1; *CIDEA*: cell death-inducing DFFA-like effector A; *UCP2*: uncoupling protein 2; *PLIN1*: perilipin 1; *CASP3:* caspase 3; *PPARg1*: peroxisome proliferator activated receptor gamma 1; *PPARg2*: peroxisome proliferator activated receptor gamma 2; *UCP1*: uncoupling protein 1; *UCP3*: uncoupling protein 3.

**Table 4:** Alterations of bacterial phyla after bariatric surgery.

Phylum	Changes	Ref.
Firmicutes	Decrease	[91,96,97,98]
Bacteroidetes	Decrease	[96]
Actinobacteria	Decrease	[96,97,98]
Chloroflexi	Decrease	[96]
Fibrobacteres	Decrease	[96]
Verrucomicrobia	Increase	[95,96]
Proteobacteria	Increase	[91,95]
Spirochaetes	Decrease	[96]
Fusobacteria	Decrease	[95,96]
